# Metabolic dysfunction-associated steatotic liver disease is associated with the risk of severe liver fibrosis in pediatric population

**DOI:** 10.1093/gastro/goaf056

**Published:** 2025-06-16

**Authors:** Wei Li, Lina Jiang, Meiling Li, Chen Lin, Li Zhu, Bokang Zhao, Yisi Liu, Yan Li, Yiyun Jiang, Shuhong Liu, Ping Liang, Junqi Niu, Jingmin Zhao

**Affiliations:** Department of Pathology and Hepatology, The Fifth Medical Center of Chinese PLA General Hospital, Beijing, P. R. China; Department of Pathology and Hepatology, The Fifth Medical Center of Chinese PLA General Hospital, Beijing, P. R. China; Department of Pathology and Hepatology, The Fifth Medical Center of Chinese PLA General Hospital, Beijing, P. R. China; Medical School of Chinese PLA, Beijing, P. R. China; Department of Pathology and Hepatology, The Fifth Medical Center of Chinese PLA General Hospital, Beijing, P. R. China; Department of Hepatology, The First Hospital of Jilin University, Changchun, Jilin, P. R. China; First Department of Liver Disease Center, Capital Medical University, Beijing, P. R. China; Department of Pathology and Hepatology, The Fifth Medical Center of Chinese PLA General Hospital, Beijing, P. R. China; Department of Pathology and Hepatology, The Fifth Medical Center of Chinese PLA General Hospital, Beijing, P. R. China; Department of Pathology and Hepatology, The Fifth Medical Center of Chinese PLA General Hospital, Beijing, P. R. China; Department of Interventional Ultrasound, The Fifth Medical Center of Chinese PLA General Hospital, Beijing, P. R. China; Department of Hepatology, The First Hospital of Jilin University, Changchun, Jilin, P. R. China; Department of Pathology and Hepatology, The Fifth Medical Center of Chinese PLA General Hospital, Beijing, P. R. China

**Keywords:** metabolic dysfunction-associated steatotic liver disease, non-alcoholic fatty liver disease, fibrosis, pediatric, liver biopsy

## Abstract

Metabolic dysfunction-associated steatotic liver disease (MASLD) has been proposed to replace the term of non-alcoholic fatty liver disease (NAFLD). To investigate the effect of MASLD on liver fibrosis and validate the clinical utility of MASLD criteria, differences in disease severity and clinical outcomes between MASLD and NAFLD were compared in a biopsy-proven pediatric cohort. The retrospective clinical data of 427 children with biopsy-proven steatotic liver between 2010 and 2021 were consecutively collected and categorized into three distinct subgroups of MASLD-only, NAFLD-only, and MASLD–NAFLD according to the diagnostic guidelines. Patients with MASLD-only and MASLD–NAFLD had more features of metabolic disorders, with higher level of triglycerides but lower level of high-density lipoprotein cholesterol than NAFLD-only. The proportion of significant fibrosis was highest in MASLD-only patients (68.0%), followed by those with MASLD–NAFLD and NAFLD-only (43.3% and 19.4%, respectively; *P *< 0.001). More steatohepatitis was presented in MASLD–NAFLD group than the other two groups (66.1% vs 30.8% vs 22.6%, *P *< 0.001). Multivariate regression revealed that children with MASLD-only had 5.8-fold greater risk of significant fibrosis than those with NAFLD-only (*P *= 0.001). After a median follow-up of 83 months, 14 of 427 patients developed clinical outcomes. Kaplan–Meier curves indicated no difference in the cumulative incidence of clinical events between the groups (log-rank, *P *= 0.073). Children in MASLD group tended to have concomitant with severe liver fibrosis and related metabolic diseases compared to those with NAFLD-only in pediatric cohort. Thus, the redefinition of MASLD may improve the detection of children with severe disease that need early intervention.

## Introduction

Metabolic dysfunction-associated steatotic liver disease (MASLD) is one of the most common chronic noninfectious liver diseases with a global prevalence of 38% in adults and up to 7%–14% in children and adolescents [1]. The rapid rise of childhood obesity prevalence has led to a parallel increase in the epidemic of MASLD [1–3], and it has been recognized that metabolic dysfunction plays a dominant role in disease pathogenesis [1, 4, 5]. Despite MASLD children are typically asymptomatic, they can also progress to advanced liver fibrosis, liver cirrhosis, hepatocellular carcinoma, and situation that needs liver transplantation [6–10], resulting in an increasing liver-related mortality and a substantial healthcare burden. Therefore, early detection and monitoring of the high-risk progression followed by appropriate intervention and management are essential to halt, reverse, and even cure the disease in pediatric population.

In 2023, the nomenclature of MASLD was proposed to replace non-alcoholic fatty liver disease (NAFLD) and metabolic dysfunction-associated fatty liver disease (MAFLD), and the consensus introduced a set of diagnostic criteria [11]. Different from NAFLD, the diagnosis of MASLD does not concentrate on exclusion of alcoholism or other causes of steatotic liver disease (SLD), whereas the presence of obesity, diabetes, hypertension, hypertriglyceridemia, or low level of high-density lipoprotein (HDL) is required for MASLD [11]. Patients with steatotic liver disease (SLD) and excessive alcohol consumption were classified as MetALD, whereas those with other specific etiologies were designated as etiology-specific SLD (e.g., drug-induced SLD or cryptogenic SLD)[11].

Multiple studies have proven the clinical applicability and efficiency of the MASLD nomenclature. Data from adults showed that the MASLD criteria identified more patients than the NAFLD criteria; the excess part is mostly patients with excessive alcohol drinking and secondary causes of SLD [12]. Another study showed that MASLD and NAFLD identified similar patients with similar clinical features [13]. The discrepancy was attributed to differences of grouping, mainly on patients with excessive alcohol drinking and those with secondary etiologies of SLD. The pediatric population have unique genetic and epigenetic susceptibilities, differences in predominant drivers, and a distinct histological pattern [1, 6, 8], while adult population and pediatric population has entirely different epidemic causes of SLD; thus, it is essential to study the effect of SLD nomenclature on pediatric patients. Moreover, previous study showed that MASLD was linked to higher all-cause mortality risk than NAFLD patients [14]. But MASLD progression in pediatric patients still remains unclear.

Liver biopsy remains the gold standard for diagnosing SLD [15]. Significant fibrosis (stage ≥ 2) and steatohepatitis are two main histological landmarks of worse hepatic outcomes and liver-related mortality [16–19]. Determining the association of MASLD with fibrosis and steatohepatitis may help to evaluate the clinical utility of this newly proposed definition. Furthermore, few studies to date have evaluated the clinical outcomes between patients with MASLD and with NAFLD in the pediatric cohort. Therefore, we investigated the differences between MASLD and NAFLD on the disease severity and clinical outcomes in a biopsy-proven pediatric cohort. We also validated whether the MASLD would help improve the identification of children with higher risk on SLD progression compared to NAFLD.

## Methods

### Study population

All consecutive pediatric patients with liver biopsy between January 2010 and December 2021 were consecutively recruited with the electronic medical records from The Fifth Medical Center of Chinese PLA General Hospital (Beijing, P. R. China). The inclusion criteria were as follows: (1) aged ≤18 years and (2) evidence of steatosis on liver biopsy. The exclusion criteria were as follows: (1) no evidence of steatosis on liver biopsy, (2) unconfirmed liver disease, (3) hepatic carcinoma, and (4) lack of reliable laboratory data.

### Data collection

Baseline demographic data (age, sex, race, body mass index [BMI], medication history, and alcohol usage) and clinical manifestations (glycemic dysregulation, hyperlipidemia, hypertension, adiposity, and alcohol intake) were retrieved from electronic medical records. Laboratory tests were taken after fasting for at least 8 hours for platelet count, liver biochemistries, lipid profiles, plasma glucose, uric acid, and creatinine. All these assessments were detected by standard laboratory methods.

### Liver histology

The liver biopsy specimen was stained with hematoxylin-eosin, Masson’s trichrome, and Sweet’s reticulin stain and analyzed by two experienced pathologists blind to the clinical information. Histological features including the degree of steatosis, activity of necroinflammation, the presence of ballooning degeneration, and the stage of fibrosis were assessed by nonalcoholic steatohepatitis Clinical Research Network (NASH-CRN) scoring system [20]. All specimens were reevaluated and then calculated the NAFLD activity score (NAS) for steatosis (0–3), hepatocyte ballooning (0–2), and lobular inflammation (0–3).

### Definition of NAFLD and MASLD categories

SLD was determined by the presence of >5% macrovesicular or microvesicular steatosis in hepatocytes when examined under light microscopy. The degree of steatosis was scored on a scale from 1 to 3 (1 = mild, <33%; 2 = moderate, 33%–66%; 3 = severe, >66%) according to NASH-CRN scoring system [20].

Pediatric NAFLD was diagnosed as having hepatic steatosis based on liver biopsy in children (≤18 years) without secondary causes of steatosis, such as viral infections, use of steatogenic drugs, genetic metabolic disorder, or excessive alcohol consumption according to NASPGHAN Clinical Practice Guideline [21].

Pediatric MASLD was diagnosed as the presence of SLD (liver biopsy) plus one of the following five conditions: (1) overweight or obesity (BMI ≥ 85th percentile for age/sex or waist circumference > 95th percentile), (2) prediabetes or type 2 diabetes mellitus (T2DM), (3) hypertension, (4) hyperlipemia, and (5) plasma HDL cholesterol (HDL-C) ≤ 1.0 mmol/L [≤40 mg/dL] or under lipid lowering treatment [11]. In this cohort, BMI percentiles were evaluated based on the American Academy of Pediatrics reference BMI for age-to-sex growth curves [22]: overweight was defined as 85th ≤ BMI < 95th percentile and obesity was BMI ≥ 95th. T2DM was defined as fasting glucose ≥ 7.0 mmol/L or anti-diabetic treatment, whereas prediabetes was defined as fasting glucose levels between 5.6 and 6.9 mmol/L or hemoglobin A1c 5.7%–6.4% [23]. Although increased waist circumference was one of the metabolic risk abnormalities, there was no data available in our cohort.

Children diagnosed with MASLD and/or NAFLD were divided into three subgroups based on their diagnostic status: (1) MASLD-only, for those who fulfilled the definition of MASLD and had chronic viral hepatitis or other secondary causes of steatosis, such as inherited metabolic disorders; (2) NAFLD-only, for those who met the definition of NAFLD with mild or no metabolic disorders; and (3) MASLD–NAFLD, for those who met both criteria for MASLD and NAFLD.

### Definition of significant fibrosis and steatohepatitis

Hepatic fibrosis was staged on a scale of F0 (no fibrosis) to F4 (cirrhosis), with F2–4 considered as significant fibrosis. Regarding steatohepatitis, we calculated the NAS and categorized patients with MASLD or NAFLD into three groups: no steatohepatitis (NAS 0–2), borderline steatohepatitis (NAS 3–4), and definite steatohepatitis (NAS ≥ 5). No and borderline steatohepatitis was combined as ‘non-steatohepatitis’. Metabolic-associated steatohepatitis (MASH) was defined as steatohepatitis with metabolic dysfunction, with or without secondary causes of other liver diseases. NASH was defined as having steatohepatitis without concomitant with other chronic liver diseases.

### Clinical outcomes

Children included in the study were then followed up at least yearly at this hospital or local hospital after the baseline biopsy. Demographic, clinical, and laboratory tests were repeated during each visit. Patient follow-up was extended until June 2022, and the primary endpoint of study was a composite endpoint, defined as the occurrence of hepatic decompensation (including jaundice, splenomegaly, and ascites), hepatocellular carcinoma, death, or other metabolic complications (including diabetic retinopathy, diabetic nephropathy, and foot ulcers).

### Ethics

The study complied with the Guideline of the 1975 Declaration of Helsinki and was approved by the ethics committee of The Fifth Medical Center of Chinese PLA General Hospital (clinical trial number: KY-2022–1-5–1). All written informed consents were obtained from parents or guardians of children before undergoing liver biopsy.

### Statistical analysis

Data were expressed as means ± standard deviation (SD) or median (interquartile range) for continuous variables and numbers (percentages) for categorical variables. The analysis of variance (ANOVA) test or Kruskal–Wallis was used to compare differences in distribution within the groups for continuous variables, and Chi-squared test or Fisher’s test for categorical variables as appropriate. Multivariate logistic regression was used to explore the association of subgroups of steatotic liver with significant liver fibrosis after adjustment for the potential clinical and demographic confounders. Cumulative incidence of clinical events was estimated using the Kaplan–Meier survival curves between the subgroups of SLD. Statistical analyses were performed in IBM SPSS software version 25.0, and *P* values were two-tailed with a significant level of < 0.05.

## Results

### Study cohort

In this single-center retrospective study, 3,176 children aged ≤18 years with liver biopsy between January 2010 and December 2021 were consecutively recruited. Of these, 2,691 children were excluded due to no evidence of steatosis on liver biopsy (*n *= 2,680), unconfirmed liver disease (*n *= 6), hepatic carcinoma (*n *= 1), or lack of reliable laboratory data (*n *= 4). In the remaining 485 individuals with complete data for assessment of MASLD and NAFLD, 427 individuals fulfilled the criteria of MASLD and/or NAFLD and enrolled in our cohort (Supplementary Figure S1). The mean age of participants at baseline biopsy was 11.2 ± 4.3 years (range from 2 to 18 years), and boys represented 81.5% of the cohort. The percentage of children with glycemic dysregulation, adiposity, hypertriglyceridemia, low HDL-C, and hypertension were 14.5%, 63.2%, 50.4%, 37.3%, and 6.6%, respectively.

### Clinical characteristics of MASLD and NAFLD categories in children

According to the corresponding criteria, 396/427 (92.7%) and 255/427 (59.7%) were diagnosed with MASLD and NAFLD of all participants with SLD, respectively (Table 1). In the cohort, 52.5% of patients met the criteria for both MASLD and NAFLD, whereas 40.3% fulfilled MASLD criteria exclusively, and the remaining 7.3% qualified for NAFLD but not MASLD. The discrepancies among three groups were shown in Table 1. Compared to MASLD-only and MASLD–NAFLD group, triglycerides level (0.89 vs 1.51 vs 1.51 mmol/L) was significantly lower whereas HDL-C was higher in NAFLD-only group. Patients with MASLD–NAFLD had a higher level of albumin (44.0 vs 41.0 g/L), TBil (9.10 vs 7.65 μmol/L), and fasting glucose (4.80 vs 4.60 mmol/L) than patients with MAFLD-only. No statistical differences were found in glutamyl transferase (GGT), alkaline phosphatase (ALP), total cholesterol (TC), and blood urea nitrogen (BUN) among three groups. Notably, cardiometabolic risk factor was more frequently found in MASLD-only and MASLD–NAFLD groups, while no metabolic syndrome was found in NAFLD-only group.

**Table 1.** Clinical characteristics of the MASLD-only, NAFLD-only, and MASLD–NAFLD

Variable	Total (*n *= 427)	MASLD-only (*n *= 172)	NAFLD-only (*n *= 31)	MASLD–NAFLD (*n *= 224)	*P*
Demographics					
Age (years)	11.2 ± 4.3	10.1 ± 4.9^a^	11.4 ± 4.3^a,b^	11.9 ± 3.6^b^	<0.001
Male, *n* (%)	348 (81.5)	124 (72.1)^a^	25 (80.6)^a,b^	199 (88.8)^b^	<0.001
BMI (kg/m^2^)	22.8 ± 5.7	20.4 ± 5.5^a^	18.9 ± 2.9^a^	25.3 ± 4.9^b^	<0.001
Comorbidity (*n*, %)					0.376
Hypertension	28 (6.6)	13 (7.6)	0 (0.0)	15 (6.7)	<0.001
Low HDL-C	159 (37.3)	64 (37.2)^a^	0 (0.0)^b^	95 (42.4)^a^	<0.001
Hypertriglyceridemia	215 (50.4)	108 (62.8)^a^	0 (0.0)^b^	107 (47.8)^c^	<0.001
Glucose intolerance	62 (14.5)	13 (7.6)^a^	0 (0.0)^a^	49 (21.9)^b^	<0.001
Overweight/obesity	270 (63.2)	83 (48.3)^a^	0 (0.0)^b^	187 (83.5)^c^	0.302
Alcohol intake	2 (0.5%)	2 (1.2%)	0 (0.0)	0 (0.0)	
Biochemistry					
Hemoglobin (g/L)	135.3 ± 14.8	131.1 ± 16.1^a^	134.5 ± 15.9^a,b^	138.7 ± 12.7^b^	<0.001
Platelet (10^9^/L)	283.0 (238.0–336.0)	274.5 (221.8–343.8)	269.0 (210.0–298.0)	291.5 (250.3–337.5)	0.067
Albumin (g/L)	43.0 (40.0–45.0)	41.0 (39.0–43.0)^a^	42.0 (39.0–45.0)^a^	44.0 (42.0–46.0)^b^	<0.001
ALT (U/L)	99.0 (61.0–149.0)	93.5 (53.0–157.3)^a,b^	78.0 (41.0–105.0)^a^	104.0 (70.3–146.8)^b^	0.007
AST (U/L)	69.0 (45.0–109.0)	76.0 (47.0–130.0)^a^	57.0 (32.0–78.0)^b^	68.5 (45.0–100.0)^a,b^	0.023
GGT (U/L)	54.0 (36.0–87.0)	53.0 (29.0–89.3)	52.0 (30.0–74.0)	56.5 (40.0–87.8)	0.187
ALP (U/L)	262.0 (160.5–343.5)	256.0 (177.3–355.8)	266.0 (120.0–351.0)	260.0 (153.3–329.3)	0.430
TBil (μmmol/L)	8.35 (6.00–12.02)	7.65 (5.53–10.9)^a^	9.90 (6.90–12.2)^a,b^	9.10 (6.40–12.90)^b^	0.008
DBil (μmmol/L)	3.10 (2.20–4.60)	2.70 (1.90–4.13)^a^	3.40 (2.40–4.50)^a,b^	3.30 (2.40–4.80)^b^	0.002
Glucose (mmol/L)	4.70 (4.40–5.10)	4.60 (4.30–4.90)^a^	4.80 (4.40–5.10)^a,b^	4.80 (4.40–5.18)^b^	0.005
HDL-C (mmol/L)	1.09 (0.92–1.31)	1.12 (0.89–1.39)^a^	1.33 (1.13–1.51)^b^	1.06 (0.91–1.18)^c^	<0.001
LDL-C (mmol/L)	2.84 (2.31–3.49)	2.76 (2.22–3.40)^a,b^	2.51 (2.06–2.89)^a^	2.98 (2.47–3.54)^b^	0.007
Triglycerides (mmol/L)	1.47 (1.03–2.04)	1.51 (1.04–2.17)^a^	0.89 (0.72–1.11)^b^	1.51 (1.15–2.04)^a^	<0.001
TC (mmol/L)	4.28 (3.67–5.00)	4.28 (3.61–5.10)	4.09 (3.62–4.51)	4.34 (3.73–4.98)	0.451
Uric acid (μmol/L)	355.0 (273.0–435.0)	282.5 (233.3–392.3)^a^	316.0 (259.0–375.0)^a^	402.0 (331.8–462.0)^b^	<0.001
BUN (mmol/L)	3.80 (3.20–4.60)	3.90 (3.20–4.70)	3.80 (3.20–4.60)	3.80 (3.10–4.50)	0.515
Creatinine (μmol/L)	51.0 (42.0–61.0)	46.0 (39.0–56.0)^a^	52.0 (44.0–57.0)^a,b^	55.0 (48.0–65.0)^b^	<0.001

Common superscript letters denote no statistically significant difference between two groups, whereas distinct superscripts indicate a significant intergroup difference (*P *< 0.05). Superscript letters (e.g. a, b, c) were used to indicate the pairwise comparison results among groups. Single superscript indicates no significant difference with other groups, while different superscripts indicate a statistically significant difference between groups.

BMI = body mass index, HDL-C = high-density lipoprotein cholesterol, ALT = alanine aminotransferase, AST = aspartate aminotransferase, GGT = glutamyl transferase, ALP = alkaline phosphatase, TBil = total bilirubin, DBil = direct bilirubin, LDL-C = low-density lipoprotein cholesterol, TC = total cholesterol, BUN = blood urea nitrogen, MASLD = metabolic dysfunction-associated steatotic liver disease, NAFLD = non-alcoholic fatty liver disease.

### Histological features of children with MASLD and NAFLD

Histologically, the NAS was significantly higher in MASLD–NAFLD group than MASLD-only and NAFLD-only groups (4.79 ± 1.21 vs 3.73 ± 1.26 and 3.55 ± 1.18, *P *< 0.001) (Table 2). Furthermore, more steatohepatitis was presented in MAFLD-NAFLD group than the other two groups (66.1% vs 30.8% vs 22.6%, *P *< 0.001). Children with MAFLD-only were more likely to have grade 2 necroinflammation or above but less likely to develop severe steatosis. In term of fibrosis, the MASLD-only group had the greatest rate of significant fibrosis (68.0%) compared to the MASLD–NAFLD group (43.3%) and the NAFLD-only group (19.4%).

**Table 2.** Comparison the histology between subgroup of MASLD-only, NAFLD-only, and MASLD–NAFLD

Variable	Total (*n *= 427)	MASLD-only (*n *= 172)	NAFLD-only (*n *= 31)	MASLD–NAFLD (*n *= 224)	*P*
Hepatic steatosis (*n*, %)					<0.001
Mild–moderate	231 (54.1)	132 (76.7)^a^	19 (61.3)^a^	80 (35.7)^b^	
Severe	196 (45.9)	40 (23.3)^a^	12 (38.7)^a^	144 (64.3)^b^	
Activity of LI (*n*, %)					0.002
G0–G1	251 (58.8)	92 (53.5)^a^	27 (87.1)^b^	132 (58.9)^a^	
G2–G3	176 (41.2)	80 (46.5)^a^	4 (12.9)^b^	92 (41.1)^a^	
Ballooning (*n*, %)	176 (41.2)	91 (52.9)^a^	19 (61.3)^a^	66 (29.5)^b^	<0.001
0 (None)	251 (58.8)	81 (47.1)^a^	12 (38.7)^a^	158 (70.5)^b^	
1 (Few)–2 (Many)	4.27 ± 1.34	3.73 ± 1.26^a^	3.55 ± 1.18^a^	4.79 ± 1.21^b^	<0.001
NAFLD activity score, mean ± SD	2.16 ± 0.86	1.72 ± 0.82^a^	2.00 ± 0.89^a^	2.51 ± 0.72^b^	<0.001
Steatosis score (0–3)	1.43 ± 0.55	1.50 ± 0.59^a^	1.13 ± 0.34^b^	1.42 ± 0.53^a^	<0.001
LI score (0–3)	0.68 ± 0.64	0.51 ± 0.58^a^	0.42 ± 0.56^a^	0.85 ± 0.65^b^	<0.001
Ballooning score (0–2)	208 (48.7)	53 (30.8)^a^	7 (22.6)^a^	148 (66.1)^b^	<0.001
Steatohepatitis (*n*, %)					<0.001
Stage of fibrosis (*n*, %)					
F0–F1	207 (48.5)	55 (32.0)^a^	25 (80.6)^b^	127 (56.7)^c^	
F2–F4	220 (51.5)	117 (68.0)^a^	6 (19.4)^b^	97 (43.3)^c^	

Common superscript letters denote no statistically significant difference between two groups, whereas distinct superscripts indicate a significant intergroup difference (*P* < 0.05). Superscript letters (e.g. a, b, c) were used to indicate the pairwise comparison results among groups. Single superscript indicates no significant difference with other groups, while different superscripts indicate a statistically significant difference between groups.

G = grade, F, fibrosis, LI = lobular inflammation, MASLD = metabolic dysfunction-associated steatotic liver disease, NAFLD = non-alcoholic fatty liver disease.

### Factors associated with significant fibrosis in pediatric SLD

Multivariable logistic regression analysis was performed to determine factors associated with significant fibrosis in pediatric SLD (Table 3). After adjustment for age, gender, hemoglobin, albumin, ALT, AST, ALP, TBil, creatinine, uric acid, inflammatory activity, and steatotic liver status, elevated AST level was independently associated with a higher risk of significant fibrosis (adjusted odd ratio [aOR] 1.007, 95% confidence interval [CI]: 1.002–1.011, *P *= 0.002), while TBil was negatively associated with significant fibrosis (aOR 0.978, 95% CI: 0.957–1.000, *P *= 0.046). Notably, children with MASLD-only had 5.8-fold higher risk of adverse fibrosis compared to NAFLD-only (aOR 5.801, 95% CI: 2.145–15.684, *P *= 0.001).

**Table 3.** Association of MASLD or NAFLD with significant fibrosis

Variable	Univariable analysis		Multivariable analysis	
	OR (95% CI)	*P*	Adjusted OR (95% CI)	*P*
Age (years)	0.946 (0.905–0.990)	0.016		
Male	1.691(1.025–2.289)	0.040		
Hemoglobin (g/L)	0.978 (0.965–0.992)	0.001		
Albumin (g/L)	0.942 (0.893–0.993)	0.026		
ALT (U/L)	1.005 (1.002–1.007)	0.001		
AST (U/L)	1.011(1.007–1.015)	<0.001	1.007 (1.002–1.011)	0.002
ALP (U/L)	1.002 (1.000–1.004)	0.015		
TBil (μmol/L)	0.975 (0.955–0.996)	0.020	0.978 (0.957–1.000)	0.046
Uric acid (μmol/L)	0.997 (0.996–0.999)	0.004		
Creatinine (μmol/L)	0.975 (0.962–0.988)	<0.001		
Significant inflammation	4.826 (3.166–7.356)	<0.001	3.928 (2.478–6.227)	<0.001
Steatotic liver status				
NAFLD-only	1.000		1.000	
MASLD-only	8.864 (3.439–22.847)	<0.001	5.801 (2.145–15.684)	0.001
MASLD–NAFLD	3.182 (1.256–8.061)	0.015	2.199 (0.826–5.851)	0.115

Multivariable logistical model was adjusted for age, male, hemoglobin, albumin, ALT, AST, ALP, TBil, creatinine, uric acid, significant inflammation, and steatotic liver status. The factors selected into multivariable analysis were determined by univariable logistic regression that reached a significance level of *P *< 0.05. Details of each model, including the unadjusted and adjusted ORs of variables, are provided in Supplementary Table S2.

TBil = total bilirubin, ALT = alanine aminotransferase, AST = aspartate aminotransferase, ALP = alkaline phosphatase, CI = confidence interval, OR = odd ratio, MASLD = metabolic dysfunction-associated steatotic liver disease, NAFLD = non-alcoholic fatty liver disease.

### Association of MASH with significant fibrosis in children with MAFLD

Among children with MASLD, the MASH group was more likely to have higher levels of BMI, ALT, AST, and GGT than those without MASH. In addition, more significant fibrosis was seen in the MASH group (63.7% vs 44.1%, *P *< 0.001) (Supplementary Table S1). By logistic regression, MASH was independently associated with higher risk of significant fibrosis compared with non-MASH. And this result was persisted after adjustment for age, hemoglobin, albumin, ALT, AST, ALP, TBil, creatinine, and uric acid (aOR 2.529, 95% CI: 1.607–3.980, *P *< 0.001) (Table 4). Similar results were also observed for NASH, which was associated with a high probability of significant fibrosis (aOR 3.432, 95% CI: 1.815–6.490, *P *< 0.001) among children with NAFLD (Table 5).

**Table 4.** Risk factors associated with significant fibrosis in children with MASLD

Variable	Univariable analysis		Multivariable analysis	
	OR (95% CI)	*P*	Adjusted OR (95% CI)	*P*
Age (years)	0.940 (0.897–0.985)	0.010		
Hemoglobin (g/L)	0.976 (0.962–0.990)	0.001		
Albumin (g/L)	0.928 (0.878–0.981)	0.009	0.922 (0.861–0.986)	0.019
ALT (U/L)	1.004 (1.002–1.007)	0.002		
AST (U/L)	1.010 (1.006–1.014)	<0.001	1.008 (1.004–1.013)	<0.001
ALP (U/L)	1.002 (1.000–1.004)	0.021		
TBil (μmol/L)	0.974 (0.953–0.995)	0.015		
Creatinine (μmol/L)	0.974 (0.961–0.988)	<0.001		
Uric acid (μmol/L)	0.997 (0.995–0.999)	<0.001	0.997 (0.995–0.999)	0.013
MASH	2.222 (1.485–3.326)	<0.001	2.529 (1.607–3.980)	<0.001

Multivariable logistical model was adjusted for age, hemoglobin, albumin, ALT, AST, ALP, TBil, creatinine, uric acid, and MASH. The factors selected into multivariable analysis were determined by univariable logistic regression that reached a significance level of *P *< 0.05. Details of each model, including the unadjusted and adjusted ORs of variables, are provided in Supplementary Table S3.

ALT = alanine aminotransferase, AST = aspartate aminotransferase, TBil = total bilirubin, ALP = alkaline phosphatase, MASH = metabolic-associated steatohepatitis, OR = odd ratio, CI = confidence interval.

**Table 5.** Risk factors associated with significant fibrosis in children with NAFLD

	Univariable analysis		Multivariable analysis	
	OR (95% CI)	*P*	Adjusted OR (95% CI)	*P*
BMI (kg/m^2^)	1.065 (1.013–1.120)	0.014	1.105 (1.031–1.185)	0.005
Hemoglobin (g/L)	0.980 (0.961–1.000)	0.047		
ALT (U/L)	1.006 (1.002–1.009)	0.003		
AST (U/L)	1.011 (1.005–1.017)	<0.001	1.007 (1.001–1.013)	0.024
ALP (U/L)	1.003 (1.001–1.005)	0.013		
Creatinine (μmol/L)	0.977 (0.959–0.995)	0.013	0.957 (0.933–0.982)	0.001
Glucose (mmol/L)	1.374 (1.003–1.880)	0.048		
HDL-C (mmol/L)	0.312 (0.114–0.849)	0.023		
NASH	4.611 (2.574–8.259)	<0.001	3.432 (1.815–6.490)	0.001

Multivariable logistical model was adjusted for BMI, hemoglobin, ALT, AST, ALP, creatinine, glucose, HDL-C, and NASH. The factors selected into multivariable analysis were determined by univariable logistic regression that reached a significance level of *P *< 0.05. Details of each model, including the unadjusted and adjusted ORs of variables, are provided in Supplementary Table S4.

BMI = body mass index, ALT = alanine aminotransferase, AST = aspartate aminotransferase, ALP = alkaline phosphatase, HDL-C = high-density lipoprotein cholesterol, NASH = nonalcoholic steatohepatitis, OR = odd ratio, CI = confidence interval.

### Prognosis of children with MASLD and NAFLD

**Figure 1.**
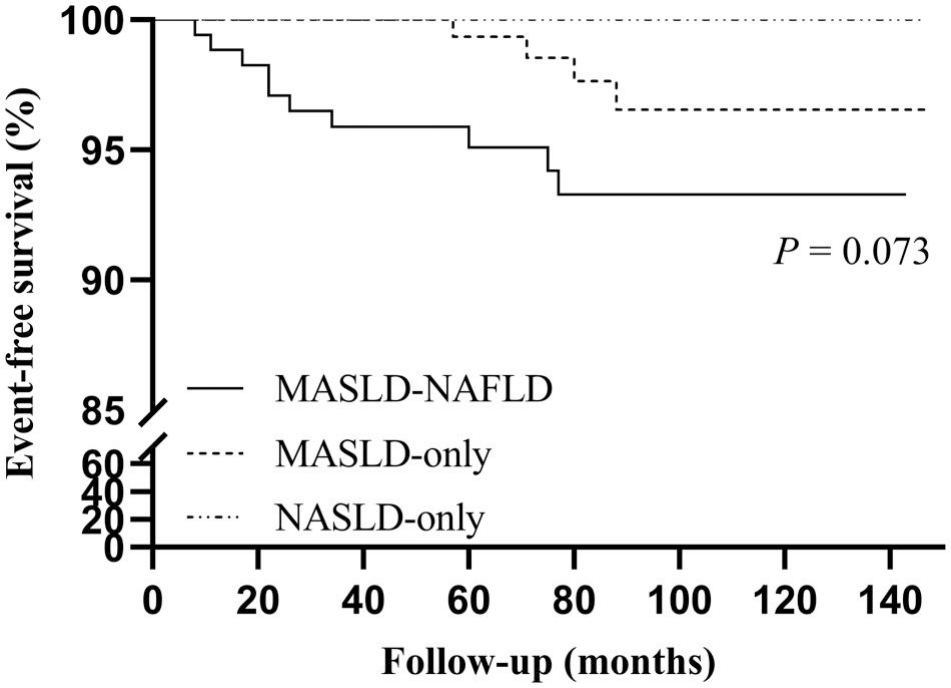
Development of clinical outcomes or death stratified by steatotic liver status.

The median follow-up was 83.0 months (interquartile range, 52.0–114.0) with a total of 2875.4 person-years. Precisely, 3.3% (14/427) patients experienced at least one clinical outcome after baseline biopsies (Table 6). One MASLD-only patient died from acute liver failure with Wilson’s disease, and another MASLD-only patient died from gastrointestinal hemorrhage with congenital liver fibrosis. The development of portal hypertension was the predominant clinical outcome in MASLD-only and MASLD–NAFLD groups. Overall, 5/172 cases of decompensated cirrhosis were observed in the MAFLD-only group and 2/224 cases in the MASLD–NAFLD group. One MASLD–NAFLD patient with baseline diabetes developed diabetic retinopathy 7 years after diagnosis. In addition, there was one case of hyperthyroidism and one case of systemic lupus erythematosus-associated nephropathy both in MASLD-only group. Kaplan–Meier survival analysis indicated no difference in cumulative incidence of event-free between the groups (log-rank test, *P *= 0.073) (Figure 1).

**Table 6.** Prevalence of first observed major adverse clinical outcomes for three groups

First event observed^a^	MASLD–only (*n *= 172)	NAFLD–only (*n *= 31)	MASLD–NAFLD (*n *= 224)
Hyperthyroidism	1	0	0
Diabetic retinopathy	0	0	1
Diabetic nephropathy	1	0	0
Foot ulcers	0	0	0
Jaundice	1	0	1
Portal hypertension^b^	5	0	2
Hepatocellular carcinoma	0	0	0
Death^c^	2	0	0
Total	10	0	4

a Clinical event was a composite endpoint, defined as the occurrence of hepatic decompensation (including jaundice, splenomegaly, and ascites), hepatocellular carcinoma, death, or other metabolic complications (including diabetic retinopathy, diabetic nephropathy, and foot ulcers).

b Portal hypertension is clinically characterized by splenomegaly and ascites.

c Two patients died as a result of acute liver failure and gastrointestinal hemorrhage.

## Discussion

Severe fibrosis is the major determinant of adverse clinical outcomes in patients with SLD, including in children [1, 4, 5, 9, 16, 17]. The redefinition of fatty liver disease (from NAFLD to SLD/MASLD) carries significant clinical and research implications, particularly for early detection and management of fibrosis in high-risk populations [24]. The World Obesity Federation (2023) projected that, without intervention, obesity prevalence will reach 25% of the global population (∼2 billion) by 2035. Childhood obesity rates may more than double, with young males rising by 100% (to 208 million) and young females by 125% (to 175 million) [1]. In this retrospective longitudinal, histology-based pediatric cohort study, we noted that children with MASLD-only and MASLD–NAFLD had worse metabolic phenotypes and histological features compared with NAFLD-only. There was a strong association between the presence of MASLD (MASLD-only) and fibrosis, and children with MASLD-only were tended to have 5.8 times higher risk of developing severe fibrosis than those with NAFLD-only. In addition, more clinical events occurred among MASLD-only patients during the follow-up period. These findings presented that the MASLD criteria was more efficient in identifying a group of patients at higher risk for disease progression compared with the traditional criteria of NAFLD and the clinical utility was also applicable to pediatric population.

Histological evidence of steatohepatitis has been proven to be a risk factor for worsening hepatic outcomes [18, 19]. Using the current scoring system of the NASH-CRN, children with MASH tended to have more significant fibrosis under biopsy in our cohort. And MASH was found to be positively associated with severe fibrosis in the MASLD group, while similar result was also observed in the NAFLD group.

Children with MASLD-only had less severe steatosis, but the proportion of significant fibrosis and pronounced necroinflammation were higher than those with NAFLD-only in this large pediatric cohort alongside detailed characterization. This result implies that the histological severity of children with MASLD is higher than NAFLD. Current research findings on liver fibrosis progression in MASLD patients remain controversial. Data from a biopsy-confirmed adult cohort also showed that SLD patients with metabolic syndrome had higher rate of advanced fibrosis and worse laboratory data compared with NAFLD [25]. A retrospective study in adult population found that the SLD with metabolic syndrome was an independent factor associated with higher liver stiffness and fibrosis by transient elastography assessment [26]. However, another study cohort demonstrated no differences in steatosis and inflammation**/**fibrosis between MASLD and NAFLD [27]. Notably, pediatric MASLD exhibits distinct clinical and pathological features versus adult cases, particularly in metabolic components and disease progression. These differences highlight the critical importance of the MASLD criteria for children, as they better reflect pediatric-specific pathophysiology. The histopathology-based pediatric cohort in our study offers greater precision in distinguishing MASLD and NAFLD. Our findings strongly support age-adapted diagnostic approaches for SLD.

We found that children with MASLD (MASLD-only) may have a higher risk of adverse fibrosis in the cohort as compared with NAFLD-only even after adjusting for potential clinical confounders. Our cohort demonstrated comprehensive liver histological features of children diagnosed with MASLD, providing robust evidence for the validity of the new definition. The possible explanation of the relationship between MASLD and adverse fibrosis might be: (a) MASLD is a hepatic manifestation of metabolic dysfunction, where systemic insulin-resistant and pro-inflammatory environment promotes the release of inflammatory cytokines such as LI-1β, IL-6, and TNF-β1 from liver immune cells [28]. Inflammatory cytokines can stimulate the proliferation of hepatic stellate cell (HSC) and the development of fibrogenesis. Mononuclear macrophages further activate stellate cells through NF-κB pathway and enhance liver fibrosis. (b) According to the definition, all children with MASLD-only in our cohort had other chronic liver diseases, such as viral hepatitis and inherited metabolic liver disease, which are known to cause hepatic fibrosis and cirrhosis [11, 18]. (c) Therefore, concomitant chronic liver diseases and metabolic components demonstrate a synergistic effect on the progression of inflammation and fibrosis, and further accelerate the development of cirrhosis and HCC [18, 29]. Based on this observation, adopting the MASLD criteria can better identify children with higher risk of significant fibrosis that are omitted by the NAFLD.

Interestingly, the NAFLD-only group seems to be more likely to have severe steatosis than the MASLD-only. As the degree of fibrosis in the MASLD-only group was more severe than that in the NAFLD-only group, it is well known that patients with advanced fibrotic SLD appear to ‘burn out’ with liver histology revealing little or no discernable fat [30]. Therefore, NAFLD-only patients had more severe steatosis than MASLD-only patients.

Additionally, MASLD criteria precisely exclude a small subset of metabolically-healthy lean children in NAFLD-only group, confirming its diagnostic accuracy.

The prevalence of clinical events was higher in MASLD-only than in MASLD–NAFLD during the follow-up period, which was mainly derived from the decompensated cirrhosis. This suggested that children with MASLD-only may have worse prognosis. The link between MASLD and increased all-cause mortality has been well established in several studies [14, 31]. SLD with metabolic dysfunction, particularly non-obese MASLD, is further proved to be an important factor associated with the presence of tumorigenesis rather than NAFLD [32]. These consistent results further confirmed that redefining MASLD will help identify patients at higher risk of complications, regardless of the presence of the secondary cause of steatosis.

To our knowledge, this is the first study to evaluate the pediatric diagnostic criteria of MASLD based on a liver-biopsy cohort and compare the clinical outcomes between MASLD and NAFLD. However, the study also consists of several limitations. First, as a single-center study, all participants came from tertiary hospital and predominantly Han ethnicity. Therefore, it may not be generalizable to all pediatric population, and population-based cohort studies are needed to confirm our findings. Second, we were unable to investigate factors associated with adverse outcomes due to relatively low number of clinical events. Large prospective studies in the future should focus on natural history to access the association between MASLD and adverse outcomes.

In conclusion, children with MASLD had more features of metabolic disorders and severe histological features. The presence of MASLD (MASLD-only) is associated with a higher risk of significant fibrosis as compared with NAFLD-only. The MASLD-only group tend to have more adverse outcomes during the follow-up period. Moreover, among children with MASLD, MASH was associated with higher risk of severe fibrosis. Therefore, the new definition of MASLD can better identify a group of patients that need early intervention and timely management. We also suggested to differentiate patients with steatohepatitis from non-steatohepatitis among MASLD.

## Supplementary Material

goaf056_Supplementary_Data
