# Supplementary material for: Metabolic dysfunction-associated steatotic liver disease is associated with the risk of severe liver fibrosis in pediatric population
Source: Gastroenterol Rep (Oxf). 2025 Jun 16;13:goaf056. doi: 10.1093/gastro/goaf056 (PMC12199907; doi:10.1093/gastro/goaf056)
Supplement: goaf056_Supplementary_Data [file goaf056_supplementary_data.docx]

**Supplementary Table 1 Clinical characteristics between MASLD/NAFLD persons with or without steatohepatitis**

| **Variable** | **MASLD** | | ***P*** | **NAFLD** | | ***P*** |
| --- | --- | --- | --- | --- | --- | --- |
|  | **MASH** | **non - MASH** |  | **NASH** | **non-NASH** |  |
| **Demographics** |  |  |  |  |  |  |
| Number, n (%)  Age (years)  Male, n (%)  BMI（kg/m^2^） | 201 (50.8)  11.3 ± 3.9  168 (83.6)  24.4 ± 5.4 | 195 (49.2)  10.9 ± 4.6  155 (79.5)  21.9 ± 5.8 | 0.504  0.293  < 0.001 | 155 (60.8)  11.9 ± 3.4  139 (89.7)  25.3 ± 4.7 | 100 (39.3)  11.9 ± 4.1  85 (85.0)  23.3 ± 5.7 | 0.652  0.264  0.001 |
| **Comorbidity（n,%）** |  |  |  |  |  |  |
| Hypertension  Low HDL-C  Hypertriglyceridemia  Glucose intolerance Overweight/obesity | 19 (9.5)  79 (39.3)  101 (50.2)  38 (18.9)  160 (79.6) | 9 (4.6)  80 (41.0)  114 (58.5)  24 (12.3)  110 (56.4) | 0.060  0.727  0.101  0.071  <0.001 | 14 (9.0)  61 (39.4)  66 (42.6)  34 (21.9)  131 (84.5) | 1 (1.0)  34 (34.0)  41 (41.0)  15 (15.0)  56 (56.0) | 0.008  0.388  0.803  0.170  < 0.001 |
| **Biochemistry**  Hemoglobin (g/L)  Platelet (10^9^/L)  Albumin (g/L)  ALT (U/L)  AST (U/L)  GGT (U/L)  ALP (U/L)  TBil (ummlo/L)  DBil (ummlo/L)  Glucose (mmol/L)  HDL-C (mmol/L)  LDL-C (mmol/L)  Triglycerides(mmol/L)  TC (mmol/L) | 136.3 ± 14.1  294.0 (253.0 – 342.5)  44.0 (41.0- 46.0)  112.0 (78.0 - 166.5)  80.0 (51.0 – 123.0)  63.0 (42.0 - 94.0)  273.0 (168.0 - 355.0)  8.35 (6.13 - 12.10)  3.10 (2.30 - 4.70)  4.80 (4.40 -5.10)  1.08 (0.90 - 1.29)  2.93 (2.41 - 3.47)  1.51 (1.15 - 2.02)  4.30 (3.69 - 4.99) | 134.4 ± 15.3  272.0 (218.0 – 336.0)  42.0 (39.0 - 44.0)  87.0 (50.0 – 128.0)  61.0 (38.0 - 97.0)  47.0 (28.0 – 73.0)  244.0 (162.0 - 331.0)  8.20 (6.00 - 11.70)  2.90 (2.10 - 4.50)  4.60 (4.40 - 5.00)  1.08 (0.89 - 1.30)  2.86 (2.29 - 3.56)  1.50 (1.04 - 2.14)  4.27 (3.66 – 5.11) | 0.199  0.005  < 0.001  < 0.001  < 0.001  < 0.001  0.190  0.640  0.366  0.211  0.685  0.940  0.952  0.932 | 138.7 ± 12.3  294.0 (254.0 – 336.0)  44.0 (42.0 - 46.0)  112.0 (78.0 - 159.0)  75.0 (50.0 - 115.0)  62.0 (43.0 - 90.0)  273.0 (164.0 - 336.5)  9.10 (6.58 - 12.68)  3.30 (2.40 - 4.80)  4.80 (4.40 - 5.10)  1.08 (0.94 - 1.22)  2.99 (2.50 - 3.50)  1.47 (1.14 - 1.96)  4.36 (3.76 - 4.98) | 137.5 ± 14.4  273.0 (228.3 – 328.0)  43.0 (42.0 - 45.0)  85.5 (52.3 - 115.8)  54.0 (36.0 - 73.0)  46.5 (29.3- 73.25)  235.0 (134.5 - 328.8)  9.35 (6.23 - 13.08)  3.25 (2.33 - 4.58)  4.70 (4.50 - 5.10)  1.09 (0.95 - 1.29)  2.80 (2.25 - 3.51)  1.34 (0.89 - 1.99)  4.10 (3.67 - 4.92) | 0.046  0.039  0.015  < 0.001  < 0.001  0.001  0.169  0.985  0.801  0.812  0.280  0.219  0.201  0.262 |
| Uric acid (μmol/L)  BUN (mmol/L)  Creatinine (μmol/L) | 384.0 (285.0 – 459.0)  3.70 (3.00 – 4.50)  53.0 (45.0 – 63.0) | 335.0 (261.0 – 427.0)  3.90 (3.30 – 4.70)  49.0 (41.0 – 62.0) | 0.005  0.017  0.064 | 402.0 (339.0 – 468.0)  3.70 (3.00 – 4.45)  55.0 (48.0 – 63.0) | 374.5 (292.3 – 434.0)  3.90 (3.30 – 4.70)  53.0 (45.5 – 66.5) | 0.012  0.081  0.493 |
| **Liver Biopsy, n (%)**  **Hepatic Steatosis**  Mild-moderate  Severe  **Activity of LI**  G0-G1  G2-G3  **Stage of fibrosis**  F0-F1  F2-F4  **Ballooning**  0 (None)  1 (Few) – 2 (Many) | 44 (21.9)  157 (78.1)  78 (38.8)  123 (61.2)  73 (36.3)  128 (63.7)  28 (13.9)  173 (86.1) | 168 (86.2)  27 (13.8)  146 (74.9)  49 (25.1)  109 (55.9)  86 (44.1)  129 (66.2)  66 (33.8) | < 0.001  < 0.001  < 0.001  < 0.001 | 25 (16.1)  130 (83.9)  67 (43.2)  88 (56.8)  72 (46.5)  83 (53.5)  17 (11.0)  138 (89.0) | 74 (74.0)  26 (26.0)  92 (92.0)  8 (8.0)  80 (80.0)  20 (20.0)  68 (68.0)  32 (32.0) | < 0.001  < 0.001  < 0.001  < 0.001 |

Abbreviations: BMI, body mass index; HDL-C, high-density lipoprotein cholesterol; ALT, alanine aminotransferase; AST, aspartate aminotransferase; GGT, glutamyl transferase; ALP, alkaline phosphatase; TBil, total bilirubin; DBil, direct bilirubin; LDL-C, low density lipoprotein cholesterol; TC, total cholesterol; BUN, blood urea nitrogen; G, grade; F, fibrosis; LI, lobular inflammation; MASLD, metabolic dysfunction associated steatotic liver disease; NAFLD, non-alcoholic fatty liver disease. MASH, metabolic associated steatohepatitis; NASH, nonalcoholic steatohepatitis.

**Supplementary Table 2 Association of MASLD or NAFLD with significant fibrosis**

|  | **Univariable analysis** | | **Multivariable analysis** | |
| --- | --- | --- | --- | --- |
|  | *OR* (95% *CI*) *P* | | *OR* (95% *CI*) *P* | |
| Age  Male  BMI  Hemoglobin  Platelet  Albumin  ALT  AST  GGT  ALP  TBil  DBil  Glucose  HDL-C  LDL-C  Triglycerides  TC | 0.946 (0.905 - 0.990)  1.691(1.025 – 2.289)  0.989 (0.956 – 1.023)  0.978 (0.965 – 0.992)  1.001 (0.999 – 1.003)  0.942 (0.893 – 0.993)  1.005 (1.002 – 1.007)  1.011(1.007 – 1.015)  1.002 (0.999 – 1.004)  1.002 (1.000 – 1.004)  0.975 (0.955 – 0.996)  0.977 (0.944 1.011)  1.040 (0.826 – 1.308)  0.755 (0.407 – 1.399)  0.992 (0.802 – 1.228)  1.134 (0.950 – 1.353)  1.016 (0.861 – 1.198) | 0.016  0.040  0.526  0.001  0.475  0.026  0.001  < 0.001  0.130  0.015  0.020  0.178  0.740  0.372  0.942  0.165  0.851 | 1.007 (1.002 – 1.011)  0.978 (0.957 – 1.000) | 0.002  0.046 |
| Uric acid  BUN  Creatinine | 0.997 (0.996 – 0.999)  0.944 (0.790 – 1.129)  0.975 (0.962 – 0.988) | 0.004  0.529  < 0.001 |  |  |
| Hypertension | 2.080 (0.919 – 4.708) | 0.079 |  |  |
| Severe Steatosis | 1.354 (0.924 – 1.985) | 0.120 |  |  |
| Significant Inflammation  Ballooning  Steatotic liver status  NAFLD-only  MASLD-only  MASLD-NAFLD | 4.826 (3.166 – 7.356)  1.456 (0.988 – 2.143)  1  8.864 (3.439 – 22.847)  3.182 (1.256 – 8.061) | < 0.001  0.057  < 0.001  0.015 | 3.928 (2.478 – 6.227)  1  5.801 (2.145 – 15.684)  2.199 (0.826 - 5.851) | < 0.001  0.001  0.115 |

**Supplementary Table 3 Risk Factors associated with significant fibrosis in children with MASLD**

|  | **Univariable analysis** | | **Multivariable analysis** | |
| --- | --- | --- | --- | --- |
|  | *OR* (95% *CI*) *P* | | *OR* (95% *CI*) *P* | |
| Age  Male  BMI  Hemoglobin  Platelet  Albumin  ALT  AST  GGT  ALP  TBil  DBil  Glucose  HDL-C  LDL-C  Triglycerides  TC | 0.940 (0.897 - 0.985)  1.689 (0.998 – 2.859)  0.975 (0.941 – 1.010)  0.976 (0.962 – 0.990)  1.001 (0.998 – 1.003)  0.928 (0.878 – 0.981)  1.004 (1.002 – 1.007)  1.010 (1.006 – 1.014)  1.002 (0.999 – 1.004)  1.002 (1.000 – 1.004)  0.974 (0.953 – 0.995)  .0974 (0.939 – 1.009)  1.014 (0.804 – 1.278)  1.045 (0.539 – 2.024)  0.927 (0.744 – 1.156)  1.057 (0.889 – 1.258)  0.988 (0.833 – 1.171) | 0.010  0.051  0.154  0.001  0.664  0.009  0.002  < 0.001  0.203  0.021  0.015  0.142  0.908  0.897  0.500  0.530  0.888 | 0.922 (0.861 – 0.986)  1.008 (1.004 – 1.013) | 0.019  < 0.001 |
| Uric acid  BUN  Creatinine | 0.997 (0.995 – 0.999)  0.940 (0.780 – 1.133)  0.974 (0.961 – 0.988) | < 0.001  0.516  < 0.001 | 0.997 (0.995 – 0.999) | 0.013 |
| Hypertension  MASH | 1.873 (0.826 – 4.249)  2.222 (1.485 – 3.326) | 0.133  < 0.001 | 2.529 (1.607 – 3.980) | < 0.001 |

**Supplementary Table 4 Risk Factors associated with significant fibrosis in children with NAFLD**

|  | **Univariable analysis** | | **Multivariable analysis** | |
| --- | --- | --- | --- | --- |
|  | *OR* (95% *CI*) *P* | | *OR* (95% *CI*) *P* | |
| Age (years)  Male, n (%)  BMI (kg/m2)  Hemoglobin  Platelet  Albumin  ALT  AST  GGT  ALP  TBil  DBil  Glucose  HDL-C  LDL-C  Triglycerides  TC | 0.939 (0.876 – 1.007)  1.249 (0.586 – 2.661)  1.065 (1.013 – 1.120)  0.980 (0.961 – 1.000)  1.002 (0.999 – 1.006)  1.038 (0.957 – 1.125)  1.006 (1.002 – 1.009)  1.011 (1.005 – 1.017)  1.001 (0.998 – 1.005)  1.003 (1.001 – 1.005)  0.964 (0.929 – 1.001)  0.930 (0.841 – 1.030)  1.374 (1.003 – 1.880)  0.312 (0.114 – 0.849)  1.101 (0.823 – 1.474)  1.053 (0.787 – 1.408)  1.018 (0.804 – 1.290) | 0.076  0.564  0.014  0.047  0.182  0.373  0.003  < 0.001  0.445  0.013  0.054  0.163  0.048  0.023  0.517  0.730  0.880 | 1.015 (1.013 – 1.185)  1.007 (1.001 – 1.013) | 0.005  0.024 |
| Uric acid  BUN  Creatinine | 0.999 (0.997 – 1.002)  0.930 (0.733 – 1.180)  0.977 (0.959 – 0.995) | 0.610  0.550  0.013 | 0.957 (0.933 – 0.982) | 0.001 |
| Hypertension  NASH | 1.744 (0.612 – 4.969)  4.611 (2.574 – 8.259) | 0.298  < 0.001 | 3.432 (1.815 – 6.490) | < 0.001 |

Children with hepatic steatosis confirmed by liver biopsy

(n=485)

All children underwent liver

biopsy from 2010 - 2021

(n=3176)

**Exclude：**

1.Without hepatic steatosis (n=2680)

2.Unconfirmed liver disease (n=6)

3.Hepatic carcinoma (n=1)

4.Missing examined data (n=4)

Children fulfilled criteria of MASLD and/or NAFLD

(n=427)

**Exclude：**

Did not fulfilled the criteria of MASLD or NAFLD (n=58)

MASLD - only

(n=172)

MASLD - NAFLD

(n=224)

NAFLD - only

(n=31)

**Supplementary Figure 1 Flow Charts of Cases Selection**
